# Clinical and radiographic evaluation of premixed versus powder/liquid bioceramic mineral trioxide aggregate in indirect pulp capping of immature permanent mandibular molars: a randomized clinical trial

**DOI:** 10.1186/s12903-026-07811-y

**Published:** 2026-02-20

**Authors:** Aisha Magdy Khairy, Rania Abdullah Taha Nasr, Maii Mohamed Ali

**Affiliations:** Pediatric Dentistry and Public Health Department, Faculty of Dentistry, Cairo University, Giza, Egypt

**Keywords:** Indirect pulp capping, Immature permanent teeth, Premixed MTA, Conventional MTA, Postoperative pain, Pulp sensibility

## Abstract

**Aim:**

This study aimed to evaluate the clinical and radiographic success rate of the Premixed MTA versus conventional powder/liquid MTA in indirect pulp capping of immature permanent carious mandibular molars.

**Methodology:**

A randomized clinical trial with a parallel group design and an allocation ratio of 1:1 was conducted at the outpatient clinic at the Pediatric Dentistry and Public Health Department, Faculty of Dentistry, Cairo University. The study included 24 pediatric patients aged 6–8 years, randomly assigned to either the premixed MTA (Well-Root™ PT) (*n* = 12) group or the conventional MTA (Cerkamed MTA+) (*n* = 12) group. Both groups underwent similar clinical procedures, including caries removal, indirect pulp capping, and restoration. Postoperative pain was assessed using a visual analogue scale (VAS). The pulp sensibility was tested using electrical pulp testing and radiographic evaluation for furcal radiolucency and root resorption using digital intraoral radiographs. Collected data were statistically analyzed at *P* < 0.05.

**Results:**

Premixed MTA group showed overall success (100%) while the conventional MTA group showed a success rate of 91.6%, at 3-month, 6-month, and 1-year follow-ups, with no significant difference between both groups (*P* > 0.05). Regarding the postoperative pain, patients who received premixed MTA showed no pain, while in the conventional MTA group, only one patient experienced pain (*P* > 0.05). No furcal radiolucency or root resorption was observed in participants from both groups during the follow-up periods.

**Conclusion:**

Both premixed MTA and conventional MTA demonstrated comparable clinical and radiographic success rates in indirect pulp capping of immature permanent molars.

**Trial registration:**

The full trial protocol and statistical analysis plan can be accessed on December 2, 2022, on the clinical trial registry page at www.clinicaltrials.gov (ID: NCT05597553), retrospectively registered.

## Background

Caries is a prevalent disease, impacting over 80% of the global population, and is a public health issue in all nations [1]. Caries induce significant direct and indirect economic expenses, and in many countries, treatment for the condition is either not covered or only partly reimbursed by public healthcare facilities [2]. Permanent first molars are highly susceptible tooth to decay due to their unique shape, function, and exposure to various environmental factors. Permanent teeth that are not fully developed often have thin roots, open apices, and roots that have not yet grown to their full length [3–5]. The young permanent tooth with deep caries lesion has an elevated risk of pulp exposure lesion during caries removal due to large dentinal tubules, pulp chamber, and prominent pulp horns [6]. Several materials are used in IPC, like calcium hydroxide (CH), which is considered the most reliable method for pulp capping because it preserves the health of the pulp, promotes the growth of new dentin, protects the pulp from damaging factors, and has antibacterial properties [7]. Nevertheless, several drawbacks have been documented throughout time regarding its use, such as inadequate sealing, insufficient chemical and mechanical bonding, weak strength, prolonged solubility, increased disintegration after acid etching, and the occurrence of tunnel flaws in the dentin bridge [8]. MTA is a bioactive, biocompatible, and antibacterial material characterized by its stability and superior sealing capability, commonly utilized as a dressing material in pulp capping procedures for permanent teeth [9]. However, many limitations were identified, including long setting time, handling properties, and tooth discoloration, making the use of this material challenging for many clinicians. To overcome these limitations, many manufacturers enhanced the MTA formulas to be premixed putty. Premixed putty are hydrophilic materials that require moisture from the adjacent tissues to set. They are ready-to-use and have superior handling properties, as well as a rapid setting time. It is advantageous for them to be less technique-sensitive, as they are less sensitive to blood contamination and moisture. They become rigid and slightly expand upon setting, thereby ensuring a superior long-term seal [10]. Up until now, limited clinical study has employed this novel material in an IPC procedure. Upon perusing the literature, it was evident that there is a lack of data and contradictory results regarding the evaluation of premixed versus powder/liquid bioceramic mineral trioxide aggregate in indirect pulp capping of immature permanent mandibular molars. Therefore, this study aimed to assess the clinical and radiographic success rates of the premixed bio-ceramic MTA versus Conventional MTA in indirect pulp capping of immature permanent carious mandibular molars. The null hypothesis (H₀) was that there would be no significant difference in clinical or radiographic success rates between premixed and conventional MTA in indirect pulp capping of immature permanent molars.

## Methods

### Study design and ethical approval

A randomized clinical trial (RCT) with a parallel group design and an allocation ratio of 1:1 was conducted in the Pediatric and Public Health Department, Faculty of Dentistry, Cairo University. The study was reviewed and approved by the Research Ethics Committee of the Faculty of Dentistry, Cairo University, on 28/3/2023, no. 2323. The full trial protocol and statistical analysis plan can be accessed on the clinical trial registry page at www.clinicaltrials.gov (ID: NCT05597553).

### Changes to trial protocol

No important changes were made to the trial protocol after recruitment commenced.

### Sample size calculation

By assuming an alpha (α) level of 0.05, and an adopting confidence interval of 95%, the effect size for intervention was 0.98 and 0.92 for the comparator, and a Beta (β) level of 0.20 (20%), i.e., power = 80%, with increased number of anticipated missing data of 20%, the predicted sample size (n) was a total of 24 cases 12 for each group. Sample size calculation was performed using Epi Info™ for Windows. No interim analyses were planned or conducted for this trial.

### Patient and public involvement

Patients or the public were not involved in the design, conduct, reporting, or dissemination plans of this research.

### Participants and data collection

Twenty-four medically fit and cooperative children, aged 6–8 years, with deep caries and reversible pulpitis affecting immature first permanent mandibular molars, were included. Inclusion criteria were: healthy children with deep carious lesions, positive response to cold sensibility testing, no spontaneous pain, and no radiographic pathology. Exclusion criteria included: irreversible pulpitis, periapical radiolucency, swelling, fistula, or non-restorable teeth. All included patients reported intermittent, mild-to-moderate pain triggered by stimuli, consistent with reversible pulpitis. Participants were randomly and equally assigned to either the intervention group (Premixed MTA (Well-Root™ PT)) or the control group (Conventional MTA (Cerkamed MTA+)). Full personal, medical, and dental histories of recruited patients were collected from the parents and the child. All clinical procedures were performed by a single, experienced pediatric dentist to minimize operator-dependent variability.

### Informed consent and assent

All participants were informed of complete information regarding the risks and benefits of the study, and written informed consent was obtained.

### Clinical procedures

An independent researcher, not involved in participant recruitment or treatment, generated the random allocation sequence using an online random number generator (http://www.random.org/). The sequence was created using simple randomization with no restrictions. The treating clinician, who enrolled the participants, sequentially opened the sealed envelopes only after the cavity preparation was complete, thereby implementing the random assignment. Recruited patients underwent clinical examination, and radiographic evaluations were performed using a digital periapical radiograph (parallel technique) with a size 2 radiographic X-ray sensor, dental X-ray machine (Owandy-RX, intraoral radiographic system, Owandy Radiology, New York, USA) with the following exposure parameters: 70 kVp, 7 mA, and 0.05 s exposure time. An online form generated a random sequence (http://www.random.org/). Patients were randomly allocated to treatment groups using a sealed envelope system. Following topical (8% benzocaine) (Ultra-care, Ultra Dent products, Inc., South Jordan, Utah, USA) and local anesthesia (2% Xylocaine with 1% epinephrine) (House Brand, New York, N.Y., USA), the tooth was isolated with a rubber dam clamps (KSK dentech, Tokoyo, Japan) and sheets (Sanctuary Dental Dam, Perak, Malaysia). A selective partial caries removal technique was employed, using a sterile low-speed round carbide bur # 4 (Meisinger Dental Burs, GmbH, Germany) and excavators (Dentsply^®^ Maillefer, Switzerland) to remove soft, wet dentin until firm cavity walls and leathery dentin on the floor were reached, taking care to avoid pulp exposure [11]. Cavity preparation ensured firm peripheral walls and a leathery dentin floor, with remaining dentin thickness ≥ 1 mm confirmed radiographically. Depending on their group assignment, a minimum 1.5 mm layer of either Premixed MTA (Well-Root™ PT, Vericom Co., Ltd., Chuncheon, Korea) or Conventional MTA (Cerkamed MTA+, Cerkamed, Stalowa Wola, Poland) (1:1 mixing ratio) was applied directly as the capping material, according to the manufacturer’s instructions [10]. The tooth was then immediately and permanently restored in a single visit [12]. The MTA was covered with a Resin-modified glass ionomer cement (RMGIC) (Fuji II LC, GC Corporation, Tokyo, Japan), application of a bonding agent, and restoration with resin composite using a selective etching technique, applying etchant only to enamel margins. Each layer was light-cured (Astralis, Ivoclar Vivadent, Schaan, Liechtenstein). Postoperative instructions included avoiding brushing the area for the first night and reporting any severe or prolonged pain or swelling. An immediate postoperative radiograph using periapical film size 2 was taken with XCP and acrylic stent for standardization. Brushing was avoided on the first night to prevent dislodgement of the setting capping material and to allow initial maturation, and to continue brushing and flossing as usual the next day [6]. Postoperative outcomes were rigorously assessed. Pain was evaluated using a Visual Analogue Scale (VAS) ranging from 0 (no pain) to 10 (worst pain) [13]. Pulp sensibility was tested clinically with an electrical pulp tester, which classified results as vital (0–39), partially necrotic (40–79), or non-vital (80+), and radiographically via digital periapical radiographs to detect pathological signs like furcal radiolucency or root resorption [14]. Clinical and radiographic follow-ups were conducted at 3, 6, and 12 months.

Postoperative outcomes were rigorously assessed. The primary outcome was the pulp capping procedure success rate at 12 months, defined as the absence of spontaneous pain, a positive response to sensibility testing, and no radiographic evidence of pathology. Secondary outcomes included postoperative pain (VAS), pulp sensibility (EPT) at each follow-up, and the presence of furcal radiolucency or root resorption.

Harms were predefined as any unintended clinical event, including postoperative pain, swelling, fistula formation, or pulp necrosis. These were assessed systematically at each follow-up appointment through clinical examination and patient/parent report.

### Blinding

Due to the nature of the interventions, the treating clinicians and participants could not be blinded to the group assignment after the envelope was opened. However, outcome assessors responsible for the clinical and radiographic evaluations at follow-up appointments were blinded to the group allocation. The data analyst was also blinded until the final statistical analysis was completed.

### Statistical analysis

Statistical analysis was performed with IBM SPSS Statistics Version 25 for Windows. All analyses were conducted on a per-protocol basis, including only participants who completed the trial according to the protocol. Missing data were not encountered as all randomized participants completed the study. No additional subgroup or sensitivity analyses were performed. The threshold for statistical significance was set at *p* ≤ 0.05 for all tests.

## Results

### Recruitment and follow-up

Participant recruitment took place between April 2023 and June 2023. Follow-up assessments were completed in June 2024. No ancillary analyses (subgroup or sensitivity analyses) were performed.

### Demographic data

**Fig. 1:**
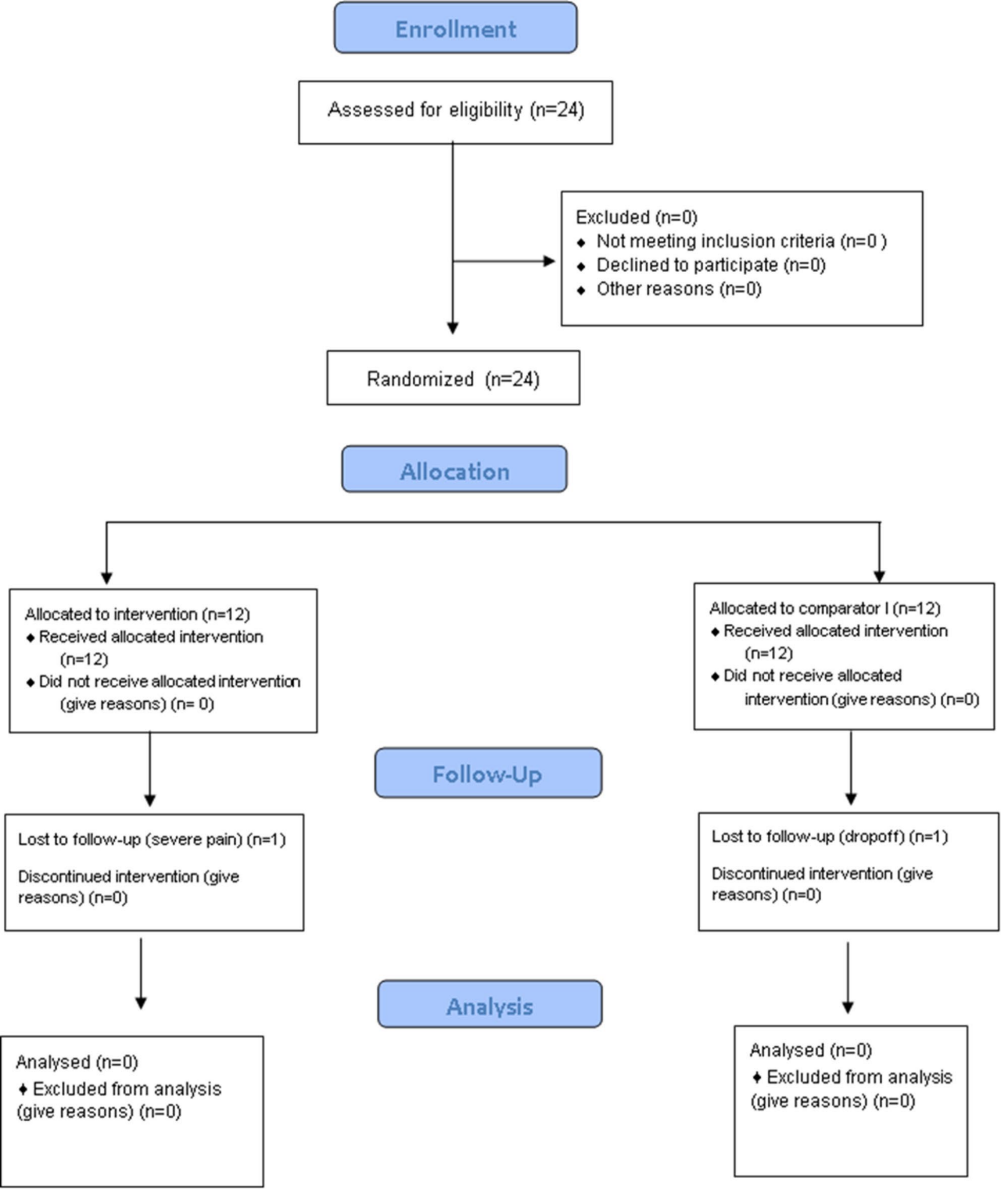
CONSORT flow diagram for the study design

There was no statistically significant difference between the two groups regarding age (*P* = 0.387). The mean age of participants in the premixed MTA group was 7.17 ± 0.58. In comparison, the mean age in the conventional MTA group was 7.42 ± 0.79 (Table 1; Fig. 1).

**Table 1:** Demographic data of participants in each group

Sociodemographic parameter		Premixed MTA	Conventional MTA	*P* value
Age (Years) (Mean ± S. D.)		7.17 ± 0.58	7.42 ± 0.79	+
Gender (n (%))	Males	7 (58.3%)	5 (58.3%)	*P* = 0.684^ns^
	Females	5 (41.7%)	7 (58.3%)	

*ns* non-significant at *P* < 0.05

### Clinical evaluation

No participants in either group reported the use of additional analgesics or received any other concomitant dental care on the treated tooth during the trial period.

### Pain level

The postoperative pain degree was measured for all patients in both groups using the VAS scale. In the group treated with premixed bio-ceramic MTA, 10 participants reported no postoperative pain, one mild pain, one moderate pain, and none experienced severe pain. In the group treated with Conventional MTA, 9 participants reported no postoperative pain, while one participant reported mild pain, one participant reported moderate pain, and one participant experienced severe pain (*P* = 0.0698) (Table 2).

**Table 2:** Testing of postoperative pain level in participants

Tested groups	Postoperative pain				P value
	No pain	Mild pain	Moderate pain	Severe pain	
Premixed MTA	10	1	1	0	0.0698^ns^
Conventional MTA	9	1	1	1	

*ns* non-significant at *P* < 0.05

Beyond the reported postoperative pain, no other harms or unintended effects, such as swelling, fistula formation, or allergic reactions, were observed or reported in either group throughout the study period.

### Sensibility test

The clinical assessment of pulp sensibility was performed throughout the 3-, 6-, and 12-month follow-up periods (Table 3.). At the baseline, all 12 participants in the premixed bio-ceramic MTA group exhibited a normal pulp sensibility response, with no negative responses. In contrast, the Conventional MTA group had 11 participants with a normal response and one; this difference was not statistically significant (*P* = 0.605). The same response in both groups was observed at 3, 6, and 12 months follow-up, and there was no statistically significant difference between the two groups at any time interval follow-up (*p* = 0.392, 0.820, and 0.348, respectively).

**Table 3.** Testing of pulp sensibility in premixed MTA and Conventional MTA groups through follow-ups

Follow-up	Premixed MTA		Conventional MTA		P-value
	Positive response	Negative response	Positive response	Negative response	
Baseline	12	0	11	1	0.605^ns^
3-months	12	0	11	1	0.392^ns^
6-months	12	0	11	1	0.820^ns^
12-months	12	0	11	1	0.348^ns^

*ns* non-significant at P < 0.05

### Radiographic findings

A radiographic assessment was carried out at the baseline at the first visit and 3, 6, and 12 months. The radiographic evaluations showed no furcal radiolucency or root resorption in both groups. (Table 4. and 5.).

**Table 4.** Furcal radiolucency for the comparison between premixed MTA and Powder/liquid MTA within each follow-up

Follow-up	Premixed bio-ceramic MTA		Conventional MTA		P-value
	No	Yes	No	Yes	
Baseline	12	0	12	0	1.00^ns^
3-months	12	0	12	0	0.067^ns^
6-months	12	0	12	0	0.131^ns^
12-months	12	0	12	0	0.089^ns^

**Table 5.** Root resorption for the comparison between premixed MTA and Powder/liquid MTA within each follow-up

Follow-up	Premixed MTA		Conventional MTA		P-value
	No	Yes	No	Yes	
Baseline	12	0	12	0	0.658^ns^
3-months	12	0	12	0	0.078^ns^
6-months	12	0	12	0	0.335^ns^
12-months	12	0	12	0	0.697^ns^

**Fig. 2:**
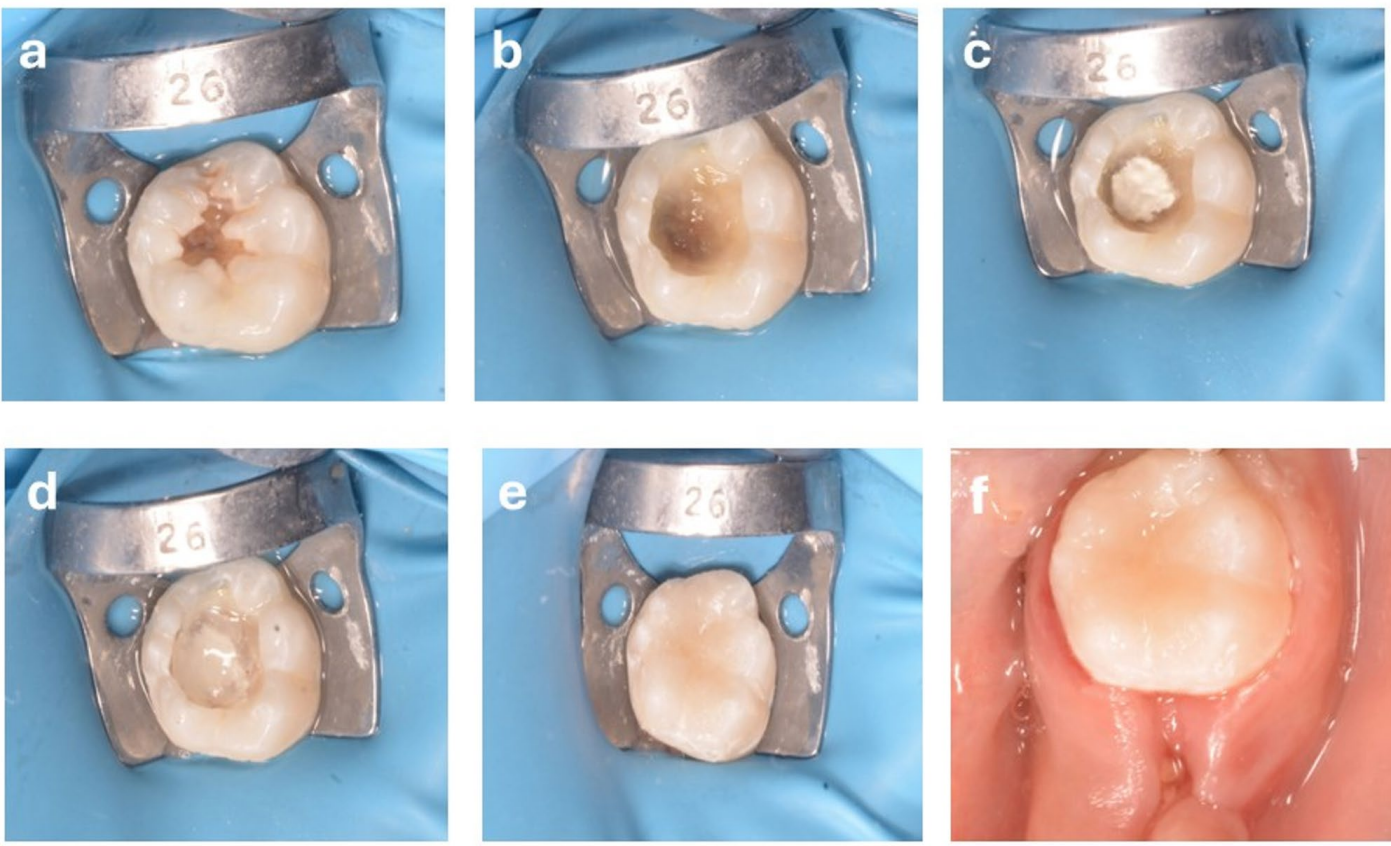
Showing lower left first immature permanent molar with deep occlusal caries (**a**) preoperative photo, (**b**) soft caries excavation done, (**c**) Powder/Liquid MTA placed into the exposed site, (**d**) resin-modified glass ionomer base given, (**e**) final restoration with resin composite, and (**f**) postoperative clinical photograph after finishing and polishing

**Fig. 3:**
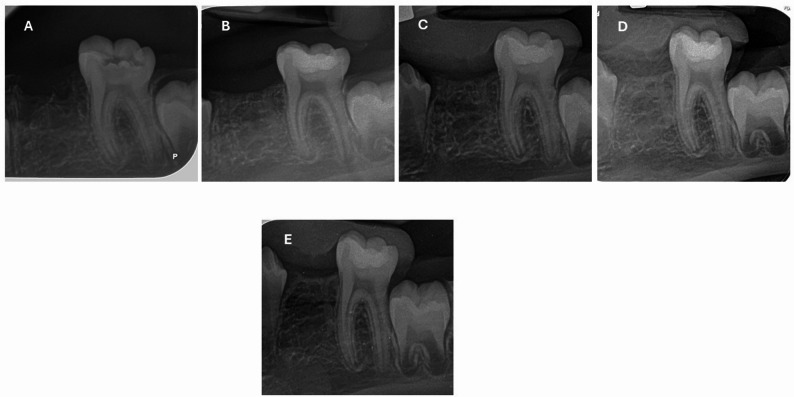
Radiographic follow-up of Powder/Liquid MTA; (**A**) preoperative, (**B**) immediate postoperative, (**C**) 3-month postoperative, (**D**) 6-month postoperative, and (**E**) 12-month postoperative

**Fig. 4:**
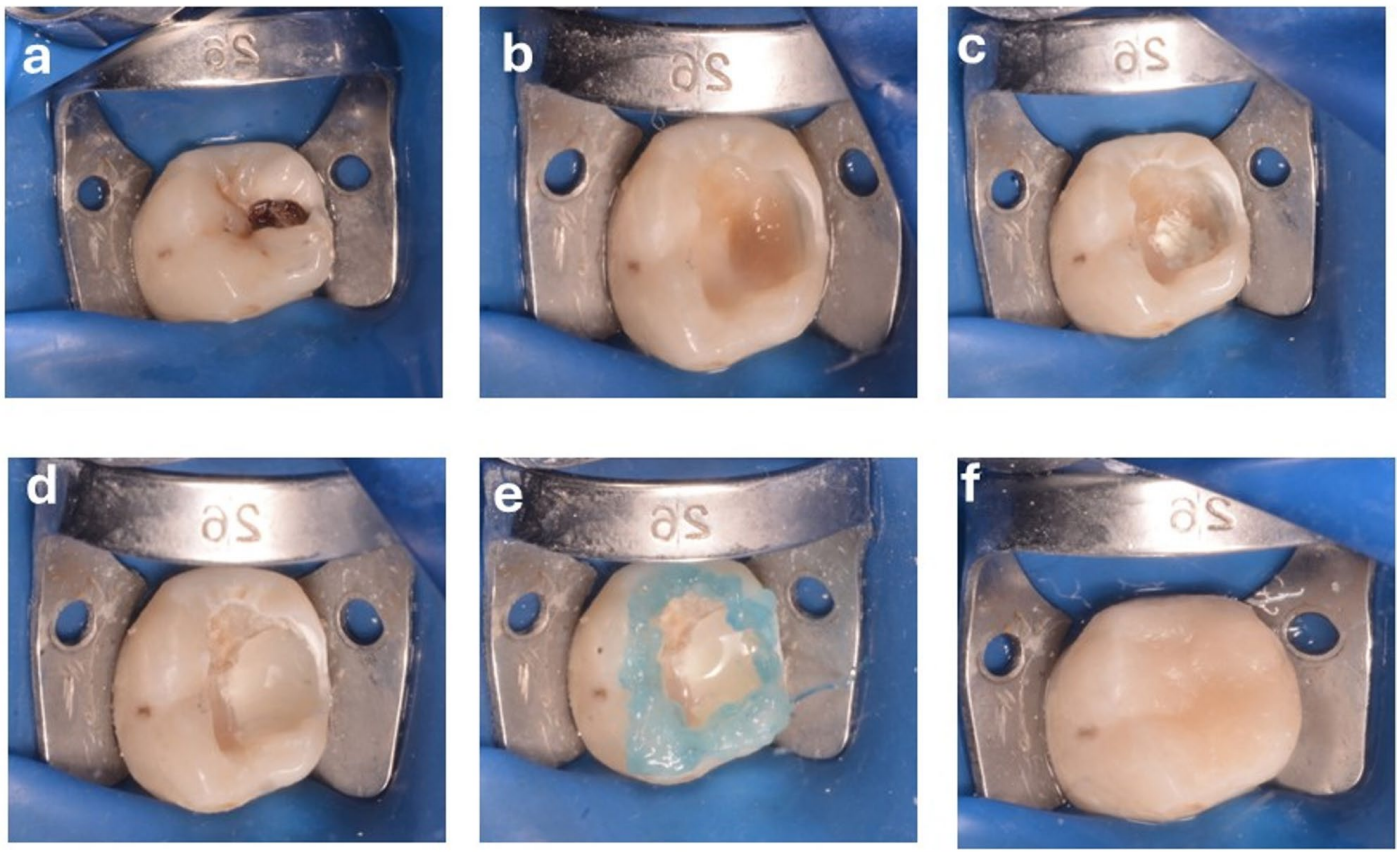
Showing lower right first immature permanent mandibular molar with deep occlusal caries, (**a**) preoperative photo, (**b**) soft caries excavation done, (**c**) premixed MTA placed into the exposed site, (**d**) resin-modified glass ionomer base given, (**e**) acid etched and final restoration with resin composite, and (**f**) postoperative clinical photograph after finishing and polishing

**Fig. 5:**
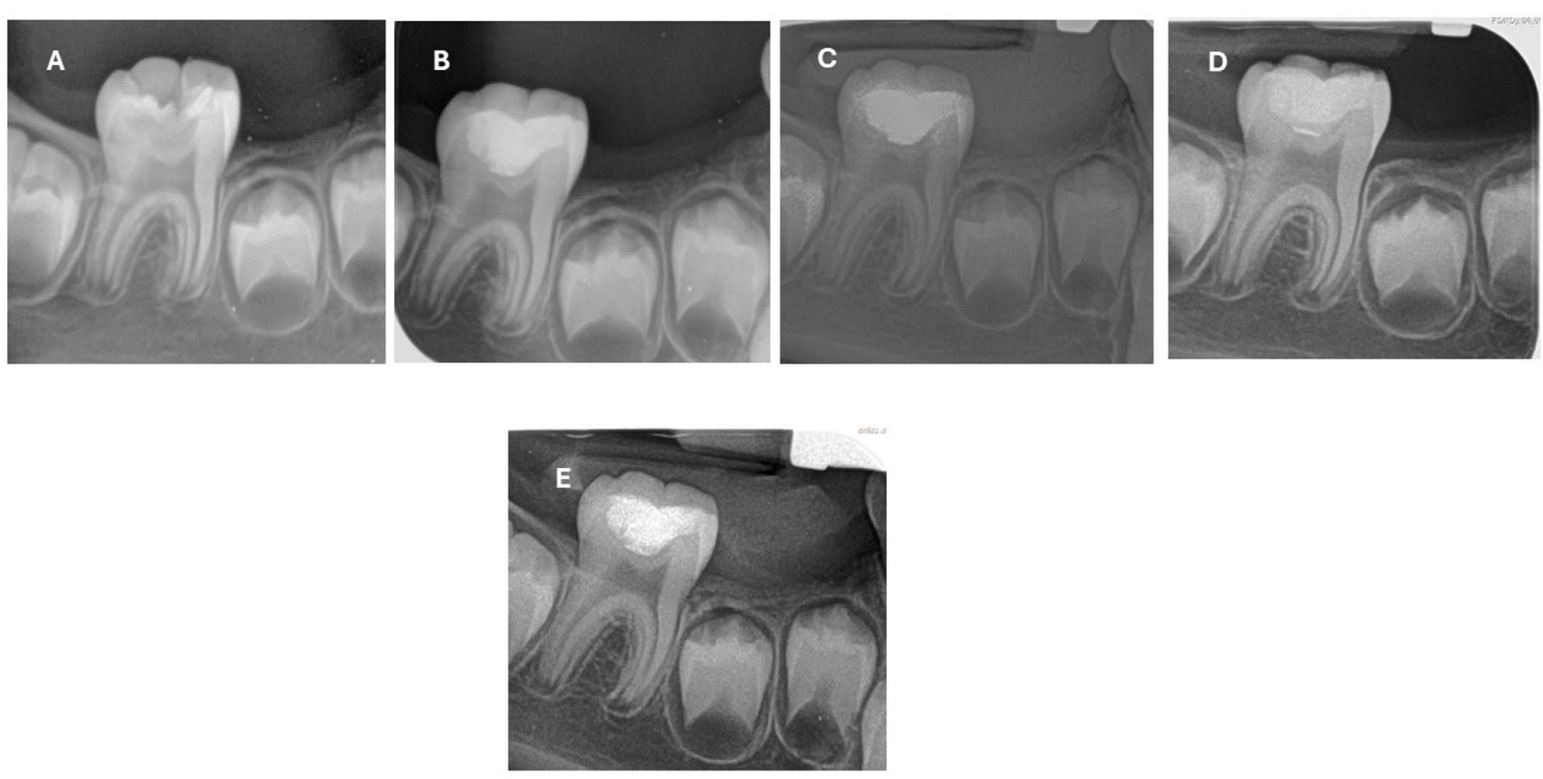
Radiographic follow-up of premixed MTA; (**A**) preoperative, (**B**) immediate postoperative, (**C**) 3-month postoperative, (**D**) 6-month postoperative, and (**E**) 12-month postoperative

The risk difference for the success rate at 12 months (Premixed MTA vs. Conventional MTA) was 8.4% (95% Confidence Interval: −9.8% to 26.6%), indicating no statistically significant difference between the groups. (Figures 2, 3, 4 and 5)

## Discussion

This randomized clinical trial (RCT) compared premixed MTA and conventional MTA for indirect pulp capping (IPC) in carious immature permanent mandibular molars. The preoperative status of the pulp influences the prognosis of indirect pulp capping. This study included only teeth with reversible pulpitis, which may have contributed to the high success rates observed. The current study was registered on ClinicalTrials.gov (www.clinicaltrials.gov) to combat reporting bias, maintain transparency, and increase confidence in clinical trials [15]. The eligibility criteria for the present study were aligned with the guidelines published by the American Academy of Pediatric Dentistry (AAPD) [11]. Indirect pulp capping (IPC) has been proposed as a conservative treatment for pulp exposure for centuries. IPC has many advantages over pulpotomy in the deepest carious layer to prevent pulp exposure, reduced cost, increased success rate, and maintenance of immature permanent primary teeth [16]. Patients aged 6 to 8 years were selected regardless of sex to ensure adequate cooperation, which may be lacking in younger age groups. This study included children with good health and no medical conditions. This decision was based on recognizing the regenerative capabilities of pulpal tissues and their susceptibility to immune responses when exposed to various biomaterials. Immature permanent teeth were selected due to their greater pulpal viability compared to mature teeth, so the pulp exhibits more responsiveness to therapy and enhanced healing capabilities due to the incomplete development of the roots of immature permanent molars. Additionally, the (XCP) alignment system was employed for a paralleling technique to facilitate comparisons of the digital radiographs [17, 18]. Vital pulp therapy involves using a rubber dam for isolation, which reduces bacterial contamination and promotes reparative dentin formation in the lesion. In this study, two materials were used: premixed MTA and conventional MTA. Premixed MTA (Well-Root™ PT) contains tricalcium silicate, dicalcium silicate, tantalum oxide, and calcium carbonate. Its setting mechanism is hydration-based, similar to conventional MTA, but does not require chairside mixing. The postoperative pain degree was measured for all patients who underwent IPC with the VAS scale. Several studies use VAS to measure and report pain after VPT, which is more sensitive than other pain scales [19]. Patients were instructed to avoid brushing the treated area on the first postoperative night to prevent mechanical disruption of the setting bioceramic material, which requires moisture and time for initial hardening. There was no statistically significant difference between the two groups, suggesting both types of bioceramic MTA were similarly effective in managing postoperative pain, with no significant advantage over the other. The VAS scale has been reported for pain assessment and is valid and reliable for children aged 8 years and older [20]. These results were similar to Roma et al. [21], who found that none of the participants reported pain at different intervals that concluding that MTA was effective in preventing postoperative pain. The study evaluated the pulp sensibility using an electric pulp tester to evaluate the pulp’s nerve response. The premixed bio-ceramic MTA group exhibited normal responses in all 12 participants at baseline, whereas the powder/liquid group had 11 normal responses and one negative response, with no significant difference, and the results remained consistent at the 3-, 6-, and 12-month follow-ups. The 12-month findings revealed no statistically significant differences in postoperative pain, pulp sensibility, or radiographic outcomes between groups, suggesting clinical equivalence in this specific context. Postoperative pain measured using the Visual Analog Scale (VAS), a validated tool for children ≥ 8 years [19, 20], was minimal and comparable across both groups. This aligns with the biological properties of MTA, which promote tissue healing through the formation of hydroxyapatite and an alkaline pH [22]. These results align with those of Roma et al. [21], who reported negligible pain after MTA-mediated IPC. The absence of significant pain in immature molars underscores MTA’s biocompatibility even in teeth with open apices and heightened inflammatory susceptibility [23]. Pulp sensibility, assessed via electric pulp testing (EPT), remained stable throughout the study. At baseline, the premixed group showed 12 out of 12 normal responses, while the powder/liquid group had 11out of 12 normal and 1 negative response (non-significant, *p* = 0.31). This initial disparity did not progress, with both groups maintaining 100% sensitivity at 12 months. These findings, in accordance with Sharma et al. [24], confirm conventional MTA’s efficacy in pulp preservation. Importantly, premixed MTA demonstrated equivalent performance, implying its ready-to-use formulation does not compromise bioactivity. Radiographic assessments (baseline, 3, 6, and 12 months) showed a complete absence of pathological features: no furcal radiolucency, internal resorption, or periapical lesions in either group. This 100% success rate at 12 months correlates with Llena et al. [25], who reported 99.4% success for IPC. The extended observation period further validates IPC as a reliable strategy for immature molars, mitigating pulp necrosis risk and supporting continued root development [26]. While Gurcan et al. [27] found comparable success for MTA, glass ionomer, and calcium hydroxide at 6 months, the study specifically confirms MTA’s sustained efficacy, irrespective of formulation, in immature teeth beyond 1 year. The demonstrated equivalence between premixed and powder/liquid MTA carries significant clinical implications. Premixed MTA eliminates the technique-sensitive mixing step inherent in traditional powder/liquid systems, mitigating risks of improper water-to-powder ratios that could compromise material properties (e.g., setting time, consistency, and sealing ability) [22]. This ready-to-use formulation streamlines the IPC procedure, particularly advantageous in pediatric dentistry, where reduced operative time enhances patient cooperation and clinical efficiency. Importantly, premixed MTA retains the bioactive advantages central to MTA’s success, including sustained alkaline pH, hydroxyapatite formation, and promotion of dentin bridge formation, without sacrificing clinical outcomes [22]. The favorable outcomes observed may be partially attributed to the strict inclusion of teeth with reversible pulpitis only, as preoperative pulp status is a well-established prognostic factor in vital pulp therapy. While both formulations effectively preserved pulp sensibility and prevented pathological sequelae in immature molars, material selection should be context-dependent. Premixed MTAs’ convenience may offset their higher material cost in high-volume or time-sensitive settings, whereas powder/liquid systems offer cost flexibility for smaller lesions. Clinicians should weigh these technical and economic factors against individual case requirements and practice logistics. However, material selection must still consider context: Premixed MTAs higher cost may be offset by operative efficiency, while powder/liquid allows incremental use in smaller lesions. The study has limitations, including a small sample size, subjectivity bias, and limited comparisons with other IPC materials. The 12-month follow-up is insufficient for long-term outcomes. Future research should focus on larger multicenter RCTs, extended observation periods, comparative studies, and objective biomarkers to improve material selection protocols and optimize IPC outcomes in young permanent dentition. Although VAS is validated for children ≥ 8 years, its use in younger children may introduce subjectivity. Future studies may consider observer-reported pain scales for younger age groups.

## Conclusion

Based on the mentioned limitations, it can be concluded that:

- Both premixed and Conventional MTA were effective materials for indirect pulp capping in immature permanent mandibular molars, as similar outcomes in terms of managing postoperative pain, maintaining pulp sensibility, and preventing radiographic pathology were obtained.
- The choice between the materials can be based on clinical preference and handling characteristics, as both offer comparable clinical efficacy.

## Data Availability

The datasets used and/or analyzed during the current study are available from the corresponding author upon reasonable request.
